# Gyroscope sensor data of shank motion during normal and barefoot walking

**DOI:** 10.1016/j.dib.2023.109858

**Published:** 2023-11-26

**Authors:** Mikko Salminen, Saku Suominen, Akihiro Inomata, Shinji Hotta, Yuki Sasamoto, Lea Saarni, Antti Vehkaoja

**Affiliations:** a Faculty of Medicine and Health Technology, Tampere University, Finland, Korkeakoulunkatu 3, 33720 Tampere, Finland; b School of Social Services and Health Care, Tampere University of Applied Sciences, Kuntokatu 3, 33520 Tampere, Finland; c Advanced Converging Technologies Laboratories, Fujitsu LTD., 4-1-1 Kamikodanaka, Nakahara-ku Kawasaki-shi, Kanagawa, 211-8588, Japan

**Keywords:** Gait analysis, Shank angular velocity, Inertial measurement unit (IMU), Gyroscope, Barefoot walking

## Abstract

In recent years, shank angular velocity (SAV) has emerged as a valuable tool for accurate temporal gait analysis and motion pattern assessment. To explore SAV among healthy subjects and its capability to distinguish differences between walking conditions, three-dimensional SAV data was measured with a gyroscope sensor during normal and barefoot walking. The resulting dataset contains measurement data from 58 healthy adult subjects aged 19 to 75 years. A single gyroscope was positioned on the lateral side of both shanks just above the lateral malleolus. The data collection involved the subjects walking a 10 m distance three times, both wearing shoes and barefoot. The subjects were instructed to walk with their own natural walking velocity, and each walk began from a stationary position. The dataset has the potential to provide information on how height and weight affect gait kinematics and how barefoot walking differ from walking with shoes. The data also supports designing the collection protocol for more extensive datasets of IMU-based shank motion during gait.

Specifications Table

**Table:** 

Subject	Orthopaedics, Sports Medicine and Rehabilitation
Specific subject area	Gait analysis based on gyroscope sensor data
Data format	Raw gyroscope sensor data, Data tables, Data analysis scripts
Type of data	Time series sensor data, Table, Signal processing code
Data collection	Data were collected using gyroscope sensors controlled by a gait analysis system. Single gyroscope sensors were attached on the lateral side of the shank of both legs, just above the lateral malleolus. Subjects were instructed to walk a 10 m walkway three times first with shoes and then barefoot. Each walk began from a stationary position. Instruments: Suunto Movesense IMU sensor Fujitsu KIDUKU Walking Monitoring gait analysis system
Data source location	Institution: Tampere University of Applied Sciences City: Tampere Country: Finland
Data accessibility	Repository name: Zenodo Data identification number: 10.5281/zenodo.7756444 Direct URL to data: https://zenodo.org/record/7756444#.ZC5tPXZBy5c

## 1 Value of the Data


 

• Gyroscope-based shank motion is used increasingly in gait studies. This dataset is collected in stable conditions according to a strict protocol to verify the quality of the data. It represents a versatile sample of healthy adult gait. The dataset includes measurements from barefoot walking and walking with shoes collected with the same protocol during the same session.
• Gait researchers who study shank motion during gait or barefoot walking vs. walking with shoes can benefit from this dataset.
• Scientists, who are developing new SAV-based methods, such as gait event detection, can benefit from this dataset.
• The dataset serves as a valuable resource for testing algorithms, getting a general idea of SAV in a versatile adequately sized sample alone or combined with other datasets collected with a similar method.
• Data can be used to explore the differences between barefoot walking and walking with shoes and differences between left and right lower limb gait patterns.
• The dataset also holds potential for future research into how subject characteristics, like height and weight, influence gait and shank motion.

## 2 Data Description

The dataset consists of the following documents and files:

1. Data description.txt: Text document describing the contents of the other files in the repository.
2. subject_measures.csv: Csv file containing information on subject weight, height, age and gender.
3. sensors.csv: Csv file linking each session and foot to the sensor ID used in measuring data.
4. GaitSAV_shoe.zip & GaitSAV_barefoot.zip: compressed folders, each containing 116 subject and foot specific csv files of gyroscope raw data from walking with shoes and barefoot, respectively. The total number of gait cycles recorded for all subjects was 3865, from which 1848 were with shoes and 2017 barefooted.
5. GaitSAV_descriptives.py: Python script for defining gait cycles out of raw data csv files and creating mean angular velocity figures shown in Fig. 2.
6. GaitSAV_subject_descriptives.py: Python script for creating subject characteristic plots shown in Fig. 3 out of subject_measures.csv file data.

**Fig. 1:**
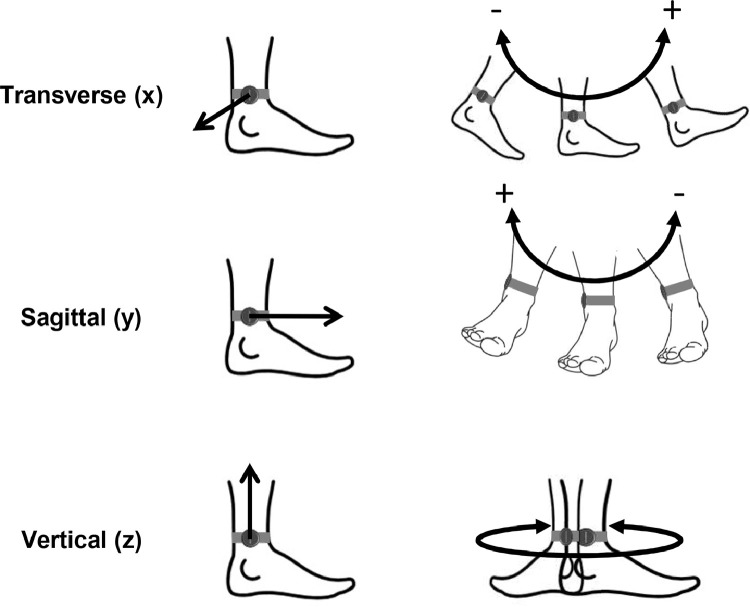
Illustrations of transverse, sagittal and vertical rotation axes of shank including defined rotation directions. For vertical rotation the axis direction depends on the leg.

The measurement data, available in GaitSAV_shoe.zip and GaitSAV_barefoot.zip, illustrates the velocity of shank angle alteration in three dimensions. Measurements are easier to understand when one identifies the plane where the shank rotation is observed. The transverse rotation axis (x) movement is observed from the side, sagittal rotation axis (y) from front or back and vertical rotation axis from above (Fig. 1).

The sensor is positioned on the lateral side of the shank, just above the lateral malleolus, as indicated in Figs. 1 and 4. To achieve the correct alignment, the sensor's vertical axis should parallel the longitudinal axis of the shank segment, while the sagittal axis should align with the walking direction, following the imaginary line connecting the back of the heel and the second metatarsal head.

**Fig. 2:**
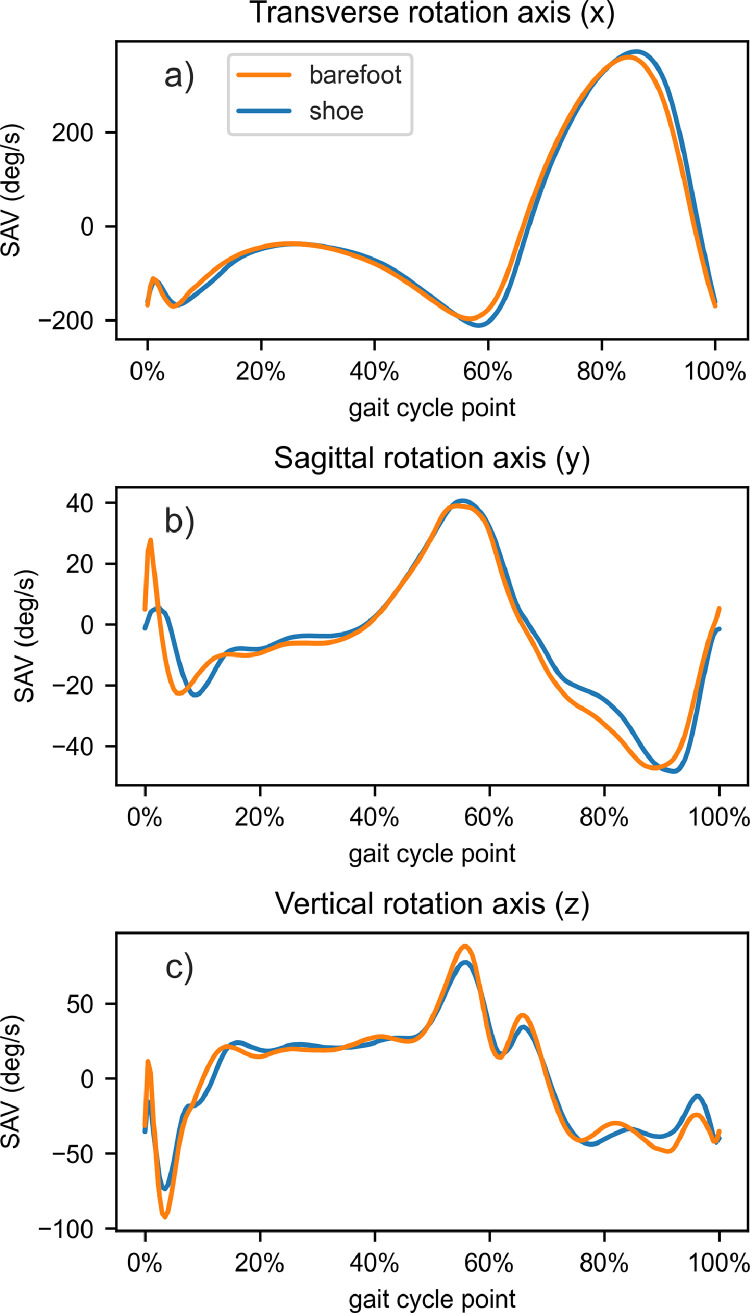
Mean SAV of all subjects during gait cycle, comparing barefoot (in orange) and shod (in blue) walking for a) transverse, b) sagittal and c) vertical rotation axes. Right leg transverse rotation axis data is multiplied by -1 to combine right and left foot data in the same plot.

The time normalized data to represent mean angular velocities during gait cycle is shown as an example of the data in Fig. 2. Furthermore, Table 1 describes the data columns of raw data files in compressed folders and Table 2 and Table 3 do the same for subject_measures.csv and sensors.csv files, respectively.

**Table 1:** Descriptions of the columns of raw data files in compressed folders.

Column	Description
UTC	UTC timestamp
Gyroscope X	Gyroscope x-axis angular velocity signal
Gyroscope Y	Gyroscope y-axis angular velocity signal
Gyroscope Z	Gyroscope z-axis angular velocity signal

**Table 2:** Descriptions of the columns of subject_measures.csv.

Column	Description
ID	Subject ID
Gender	Subject gender: Male (M) or Female (F)
Age (years)	Subject age in years
Height (m)	Subject height in meters.
Weight (kg)	Subject weight in kg

**Table 3:** Descriptions of the columns of sensors.csv.

Column	Description
Subject	Specifies subject ID
shoe_sc	Shoe scenario (shoe/barefoot)
Foot	Right/left
Sensor	Sensor ID number of the used sensor

## 3 Experimental Design, Materials and Methods

### 3.1 Participants

**Fig. 3:**
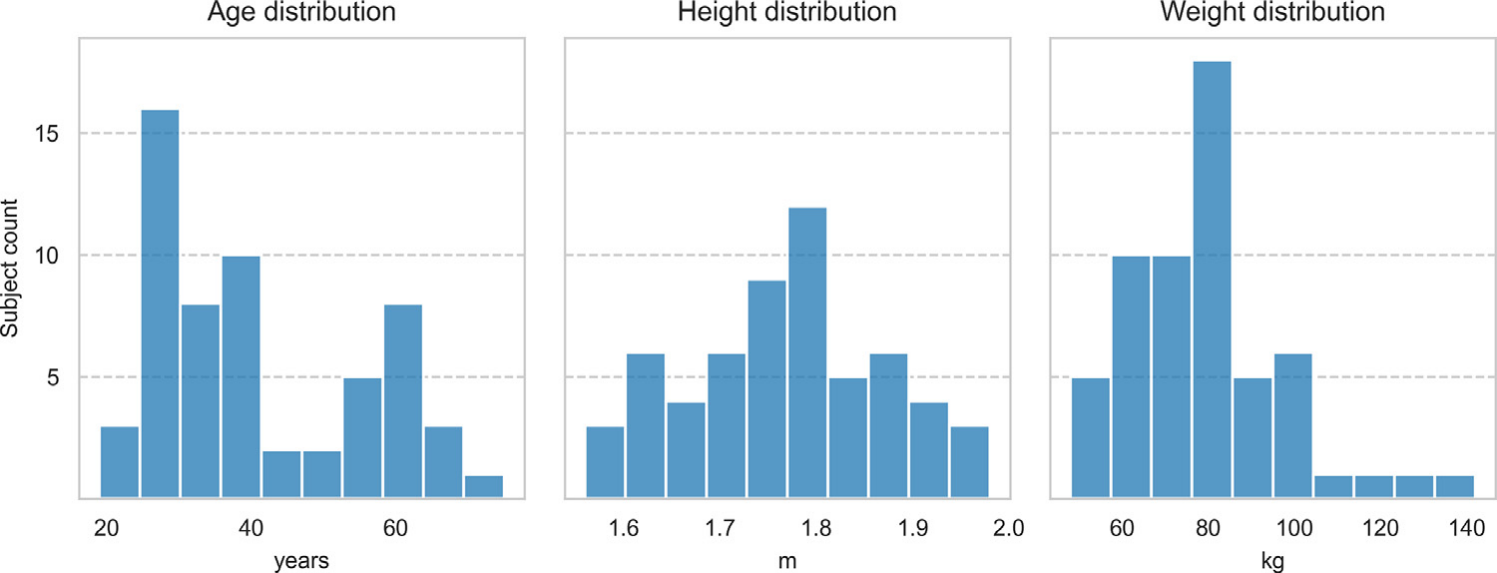
Subject age, height and weight distribution.

58 healthy adults (23 women and 35 men) were recruited for the measurements with an advert at social media and university intranet. The subjects were required to have no artificial joints, recent musculoskeletal injuries, or other disabilities that could influence their gait. Information about age, gender, body mass and body height were collected during the testing event. Information about the study and participant rights were given orally and in writing to all subjects prior to testing. All subjects gave their written informed consent to participate in the study. Subject characteristics are described more closely in Table 4 and Fig. 3.

**Table 4:** Subject characteristics.

	Age (years)	Height (m)	Weight (kg)
Mean	41	1.76	80
Std	15	0.10	18
Minimum	19	1.56	48
Maximum	75	1.98	142

### 3.2 Raw data collection

The measurements took place at the Tampere University of Applied Sciences premises. The tests were performed in closed and disturbance-free space, where an unobstructed 10 m walkway was arranged. All tests were performed on a hard floor surface with shoes that were adapt for walking (indoor sports shoes, sneakers etc.) and barefoot walking. Subjects wore clothing that did not restrict lower limb movement.

**Fig. 4:**
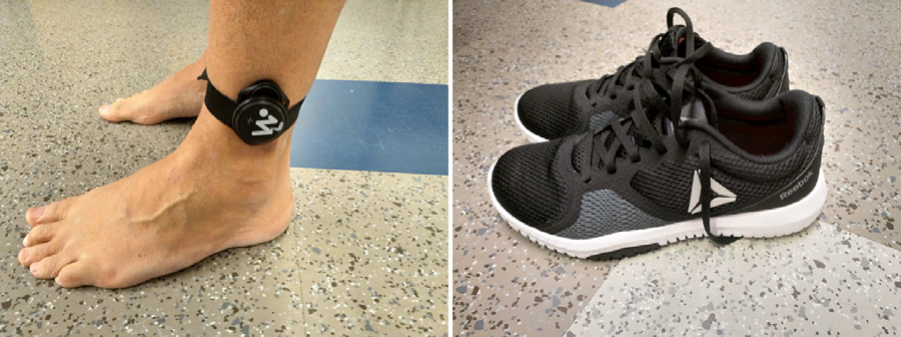
Positioning of the sensor (left) and example of the shoe type (right).

Gait data were collected using CE-marked Suunto Movesense IMU sensors (Suunto Oy, Vantaa, Finland) that utilize the LSM6DSL inertial module (STMicroelectronics Inc., Geneve, Switzerland) for gyroscope measurements. The gyroscope's accuracy is documented at ±2% [1]. The gyroscope raw data were collected with the help of CE marked IMU-based gait analysis system, Fujitsu KIDUKU Walking Monitoring, version 1 (Fujitsu Finland Oy, Helsinki, Finland). The system uses Suunto Movesense sensors, and in this case it was used to start and stop the sensor data collection. The sensors were placed with elastic straps on the lateral side of the shank above the lateral malleolus (Fig. 4). The SAV data were collected at a sampling rate of 104 Hz, which is sufficient for measuring lower limb angular velocities [2].

In each test, the 10 m walking was repeated three times first with shoes and then barefooted. Before testing, the procedure was demonstrated, and the sensors were carefully positioned to correct locations. The subjects were instructed to walk with their own natural walking velocity and to begin each 10-m walk from a completely stationary position.

### 3.3 Dataset and scripts

The dataset is stored in the Zenodo data repository with the name “Gyroscope sensor data of shank motion during normal and barefoot walking” [3]. It has previously been employed in [4]. Notably, the dataset encompasses essential scripts, namely GaitSAV_descriptives.py and GaitSAV_subject_descriptives.py, which were used to create Fig. 2 and Fig. 3 in this article. The data analysis has been made with Python 3.10. Running the codes requires installing pandas, seaborn and scipy packages.

All recorded data is accessible in the repository. Each data file in “GaitSAV_shoe.zip” and “GaitSAV_barefoot.zip” provides continuous measurement data commencing from a stationary position before the first walk and concluding at the stationary position after the third walk. Notably, the script “GaitSAV_descriptives.py” is especially valuable for data analysis, as it facilitates the extraction of gait cycles. Heel strikes are detected as the first local minima following a zero-crossing with a negative slope (ZN), provided there is a preceding zero-crossing with a positive slope (ZP) at least 150 ms before the ZN, and the maximum angular velocity between them exceeds 200 deg/s [5]. Each heel strike must also be at least 0.5 seconds apart from the previous one, with two consecutive heel strikes defining a gait cycle if the time between them is no more than 1440 ms. It should be noted that these thresholds are fine-tuned for this specific dataset and protocol and may not work optimally with other similar data.

Finally, the “cycles” dataframe at the end of the script offers data on cycle start, cycle end, cycle time, walk number, and cycle number, which can be easily exported as CSV or Excel file. This information proves valuable particularly for tasks such as excluding the initial and final steps of a walk, where acceleration and deceleration effects are most pronounced.

## Limitations

The dataset has certain limitations, such as an uneven distribution of participants in terms of age and gender. Furthermore, the gyroscope's sampling rate of 104 Hz might be insufficient in some cases. Small fluctuation of effective sample rate from the nominal 104 Hz and rare occurrences of duplicate timestamps due to technical constraints are also noted. To address these issues, data interpolation, as exemplified in the “GaitSAV_descriptives.py” script, is recommended. Additionally, it's important to recognize the presence of soft tissue artifact in such measurements.

## Ethics Statement

The research was carried out according to the relevant sections of World Medical Association's Declaration of Helsinki. Informed consent was obtained from all subjects involved in the study. The attendants were free to cancel their participation at any time without giving a reason.

## CRediT authorship contribution statement

**Mikko Salminen:** Conceptualization, Methodology, Validation, Software, Visualization, Investigation, Writing – original draft. **Saku Suominen:** Conceptualization, Methodology, Validation, Investigation, Writing – original draft. **Akihiro Inomata:** Conceptualization, Methodology, Writing – review & editing. **Shinji Hotta:** Methodology, Software, Writing – review & editing. **Yuki Sasamoto:** Methodology, Software, Writing – review & editing. **Lea Saarni:** Supervision, Writing – review & editing. **Antti Vehkaoja:** Supervision, Writing – review & editing.

## Data Availability

Gyroscope sensor data of shank motion during normal and barefoot walking (Original data) (Zenodo).
